# Evolution and Mode of Action of Pulsed Radiofrequency

**DOI:** 10.5812/aapm.10213

**Published:** 2013-03-26

**Authors:** Menno E. Sluijter, Farnad Imani

**Affiliations:** 1 Department of Pain Medicine, Swiss Paraplegic Center, Nottwil, Switzerland; 2 Department of Anesthesiology and Pain Medicine, Rasoul Akram Medical Center, Iran University of Medical Sciences (IUMS), Tehran, Iran

**Keywords:** Pulsed Radiofrequency Treatment, Ablation Techniques, Anti-Inflammatory Agents, Immune System

The rationale of Pulsed Radiofrequency (PRF) was clear and simple. The mode of action of conitnuous Radiofrequency (CRF) could be explained by destruction of afferent nerve fibers on their way from a nociceptive focus to the central nervous system. In the late eighties there was a new discussion on the validity of this theory. It had been observed that an RF lesion could also be effective if it was applied peripheral to the nociceptive focus. This was in conflict with the widely accepted concept. It was therefore suspected that a second mechanism might be operational during RF application. PRF was invented to explore this possibility, with the sole purpose of finding a less destructive and equally effective technique for the application of RF to afferent pathways. This has not come true. PRF has not developed into a technique that is suitable to block afferent signals in nerves close to the electrode. It has however developed in a direction that could not have been foreseen by the inventors at that time. PRF developed into a new direction when in 2005 Teixeira found that intra-articular application of PRF could relieve pain. This was published eventually in 2008 (1) and it is confirmed by the results in larger series of patients in this issue (2). The importance of this finding goes far beyond finding another useful indication for PRF. It implies that contrary to the effects of CRF the action of PRF is not implicitly limited to an action on nerves. The immune system came into focus, suggesting that PRF could possibly influence the nociceptive process itself. An initial report on a second invention by Teixeira is published in this issue (3). Its background follows from his invention of intra-articular PRF. If PRF has a local anti-inflammatory effect it might possibly also have a general effect on the immune system if the mode of application could be adapted. The scope of this new method may have large dimensions because the immune system is involved in so many pathological conditions. This goes beyond well-known conditions like the autoimmune diseases. For example, stress and allostatic load are connected to a whole list of serious diseases such as cancer and cardiovascular disease (4, 5). The mode of action of PRF has still not been elucidated. The first attempts to find an explanation (6) were in line with the CRF concept, which was quite natural at the time. PRF was applied on a healthy nerve in the absence of a painful condition. It was found that the application of PRF to a DRG elicits the expression of c-fos in the dorsal horn. The importance of this finding for the clinical effect of PRF is still a subject of discussion. C-fos is an aspecific marker indicating cellular activity. The suggestion that Long Term Depression (LTD) of higher afferent synapses could have developed was hypothetical. The meaning of the dorsal horn activity was never cleared up and the discussion therefore ended undecided. The alternative of modulation is clearly ablation, and in the vacuum that had persisted this possibility came up (7). Tissue destruction does occur during PRF (8-10). Theoretically this could be due either to overheating or to exposure to electric fields. The thermal effects are limited because the temperature falls off rapidly away from the electrode tip and because the shaft does not heat up at all (11, 12). A more likely candidate for causing destruction is exposure to electric fields. From Cabana’s work (13) it can be deducted that for a PRF duty cycle cell death occurs at field strength from about 10.000 V/m upwards. The maximal fields around the tip are particularly high, approximately 180.000 V/m when the customary 45 V is applied (11) and this is far into the lethal range. But just like the temperature the field strength falls off precipitously away from the electrode. The electric fields around the shaft start less spectacularly at around 50.000 V/m but they are much more persistent away from the electrode. These fields are a likely candidate for causing the reported destruction. But this narrow zone of destruction around the shaft cannot explain the mode of action of PRF. That could only be suspected if the electrode is parallel and closely adjacent to the target structure. This does not apply, for example in DRG procedures and in intra-articular procedures. A minimal ablative effect may therefore occur in special electrode positions, but explaining the mode of action of PRF in general terms by ablation is a bridge too far. An explanation of the mode of action should preferably be universal. The role of transcutaneous PRF then becomes particularly important. The efficacy of this method has now been shown in an RCT (14) and the elicited fields can be estimated to be approximately 500 V/m. Do such low fields have a biological action? Yes, they do. This is known from Cahana’s work (13). The authors studied action potentials elicited in hippocampal slice cultures, studying the effect of both PRF and CRF at 42 0C. For CRF the source voltage must have been around 9 V but this caused a clear depression of the action potentials. At such a low voltage the elicited fields may seem inconsequential, unable to mediate the long lasting, stable improvement that characterizes the effect of PRF in successful cases. Indeed, a direct effect is unlikely, but an intermediate role is conceivable. Recently it has been reported that exposure of monocytes to PRF at a field strength of 500 V/m and even lower causes expression of TNFα (15). This may of course be an unrelated event, but it could also be instrumental in a further trajectory. This trajectory is still unknown. There could be a local – or regional – effect on resident immune cells, or alternatively the afferent vagus nerve could be involved, activating the cholinergic anti-inflammatory tract (16). This would be concordant with experimental work suggesting the enhancement of noradrenergic and serotonergic descending pain inhibitory pathways following PRF treatment (17). The vagus nuclei have important connections to these tracts. Whatever happens, the immune system is a complex system, and it is known that such systems can make significant and stable changes by moving to a new attractor (18). A good example-but in the other direction-is the change in phenotype from active to regulatory of the invariant NKT lymphocytes following a stroke, causing the high mortality from pneumonia in these patients (19). This shows how drastic these changes may be. Much of what happens after PRF application is still in the clouds, but the contours are slowly getting clearer.
